# A Missed Meal, A Missed Diagnosis: Why Emergency Departments Must Lead on Food Insecurity Screening

**DOI:** 10.5811/westjem.47454

**Published:** 2025-07-10

**Authors:** Victor Cisneros, Ian Olliffe, Raymen R. Assaf

**Affiliations:** * Eisenhower Health, Department of Emergency Medicine, Rancho Mirage, California; † University of California, Irvine, Department of Emergency Medicine, Irvine, California; ‡ Rady Children’s Health, Emergency Medicine Specialists of Orange County, Department of Emergency Medicine, Orange, California

**To the Editor:**

The recent recommendation by the US Preventive Services Task Force (USPSTF) concluding that there is “insufficient evidence” to assess the benefits and harms of food insecurity screening in the primary care setting may inadvertently stall momentum in addressing one of the most pressing social drivers of health: food insecurity,^1^–^4^ which affected 12.8% of US households in 2022. It disproportionately impacts Black (22.4%) and Hispanic (20.8%) families, demonstrating profound associations with adverse health outcomes, including increased number of emergency department (ED) visits, hospitalizations, worse chronic disease management, and mental health comorbidities.^1^–^2^,^5^–^6^ The ED serves as an entry point to healthcare for patients facing economic hardship^7^ and often provides the main contact some families have with the healthcare system.^8^–^9^

Each year 155 million Americans visit the ED, representing about 47% of the population, and these patients are disproportionately underinsured.^10^–^11^ Emergency physicians frequently observe the impacts of food insecurity when managing conditions such as uncontrolled diabetes or asthma exacerbations,^12^ where food insecurity significantly contributes to poor outcomes by hindering effective management, often due to resource trade-offs between food and essential medications.^13^–^15^

Over the past three years, we have led feasibility studies and implemented screening across adult and pediatric EDs. We found that 21.8% of caregivers screened positive for food or housing insecurity in a pediatric ED.^16^ In an adult ED, 16.9% of patients reported food insecurity.^17^ Furthermore, findings from our adult ED study—in which the participants we followed showed improved food security scores after receiving resource information—support the plausibility of ED-based interventions helping to alleviate food insecurity.^17^

The ED serves high volumes of underinsured, unhoused, and high-acuity patients.^7^,^18^ Preventive care gaps are the norm, and the ED often functions as the default site for both clinical and social triage.^8^,^19^

Emergency department-based screening tools can identify food insecurity among patients not captured through primary care screening; these include individuals without a primary care physician whose housing may be sporadic or who are living in resource deserts. The ED is far more than a safety net; it mirrors the state of community health, where upstream failures surface downstream with consequences of poorer health incomes and higher healthcare costs. In contrast, the evidence gap cited by the USPSTF reflects the known structural limitations in that setting: variable visit frequency; under-resourced clinics, and reimbursement models that do not support social screening.^1^,^20^

We call on healthcare leaders, policymakers, and emergency physicians to consider the ED not as a place where food insecurity screening is “optional,” but where it is **essential**. Federal and state policy should incentivize ED-based screening workflows, fund navigator roles, and hospitals should integrate social determinants of health into the electronic health record. Medical education and residency training programs must prepare future clinicians to view food insecurity as an integral component of healthcare.
